# Analyzing diversity, equity, and inclusion content on dermatology fellowship program websites

**DOI:** 10.1080/10872981.2024.2347762

**Published:** 2024-05-01

**Authors:** Forrest Bohler, Allison Garden, Varna Taranikanti

**Affiliations:** a Medical Student, Oakland University William Beaumont School of Medicine, Rochester, MI, USA; b Medical Student, Edward Via College of Osteopathic Medicine - Carolinas Campus, Spartanburg, SC, USA; c Department of Foundational Medical Studies, Oakland University William Beaumont School of Medicine, Rochester, MI, USA

**Keywords:** Diversity, equity, and inclusion, DEI, graduate medical education, dermatology, program websites

## Abstract

Diversity, Equity, and Inclusion (DEI) initiatives have garnered increasing attention within medical education as there have been increased efforts to diversify the physician workforce among medical students, residents, fellows, and attendings. One way in which programs can improve their DEI initiatives and attract a more diverse pool of applicants is through DEI content on their graduate medical education websites. Prior studies characterizing the content and prevalence of DEI material on residency webpages have shown that dermatology residencies have relatively low levels of DEI content on their websites in which almost ¾ of all programs having no DEI content. Little is known, however, if similar findings are to be expected for the three main dermatology subspecialty fellowship program webpages: Dermatopathology, Pediatric Dermatology, and Micrographic Surgery and Dermatology Oncology. Fellowship programs were identified using the Accreditation Council for Graduate Medical Education’s online database of fellowship programs. Programs were evaluated on a standardized scoring system for five equally weighted criteria: fellowship-specific DEI webpage, DEI commitment statement, DEI initiatives (summer research opportunities for under-represented minorities, DEI council, etc.), link to the institution’s DEI homepage, and information about bias training. The mean score among all programs was 12.5. Pediatric dermatology ranked the highest among all specialties, while Mohs ranked the lowest. A link to the institution’s DEI homepage was the most prevalent factor accounting for 42.1% of all programs collected, whereas information about bias training and fellowship-associated DEI webpage were the least prevalent. The results of this study reveal an overall lack of DEI content across all dermatology subspecialties’ webpages and represent an actionable area of improvement for fellowship directors to increase their DEI efforts to attract a diverse pool of applicants to their program.

## Background

Efforts to prioritize diversity, equity, and inclusion (DEI) to address healthcare disparities has become a central focus in medical school, residency, and fellowship programs. Findings have shown racial concordance between physicians and patients can lead to better health outcomes, reducing such disparities among marginalized populations [1,2]. Improving diversity within medicine starts with the recruitment and training of underrepresented groups in both medical school and graduate medical education. Historically, this has been accomplished, in part, through a variety of methods such as race conscious and wholistic admission processes, creating DEI committees, and increasing the visibility of under-represented minorities within their programs [3–5].

Despite these efforts, the field of dermatology ranks as the second least diverse specialty in medicine [3]. Since the COVID-19 pandemic, graduate medical education programs (GMEP) have increasingly relied on virtual resources to advertise their program to prospective medical students and residents. GMEP websites are a useful and influential resource that are utilized by prospective applicants to obtain information regarding a program’s training, culture, and overall fit [6,7]. One way in which programs can improve their DEI initiatives in order to potentially attract a more diverse pool of applicants is through DEI content on their GME websites. Findings show that visibility and messaging to prospective applicants are barriers faced by GMEPs when attempting to attract applicants from under-represented groups [8]. Given that GMEP websites are frequently utilized by prospective applicants to gain a better understanding of a program, inclusion of DEI content on GMEP webpages offer a potential solution to these barriers [6]. Despite this, prior studies demonstrate that dermatology residency programs’ websites have a dearth of DEI content [9,10]. Little is known, however, if similar findings are to be expected for dermatology fellowship programs. The American Board of Dermatology recognizes three dermatology subspecialties: Dermatopathology, Pediatric Dermatology, and Micrographic Surgery and Dermatologic Oncology (Mohs). The aim of this study is to analyze the presence and characterize the content of DEI information on fellowship websites.

## Materials and methods

Fellowship programs were identified using the Accreditation Council for Graduate Medical Education’s online database of fellowship programs [11]. Programs that did not have websites were excluded from this study (*n* = 19). A total of 152 programs were analyzed. Programs were evaluated on a standardized scoring system for five equally weighted criteria: fellowship-specific DEI webpage, DEI commitment statement, DEI initiatives (summer research opportunities for under-represented minorities, DEI council, etc.), link to the institution’s DEI homepage, and information about bias training. These criteria were derived from previous study designs that investigated similar DEI content in other medical specialties [10,12].

Data was collected independently by two separate individuals for consistency. Any discrepancies between programs’ scores were discussed openly by the authors and, if needed, voted on to reconcile any differences.

DEI content on fellowship programs’ websites was quantified utilizing a scoring system ranging from zero to 100 potential points (0 = No DEI content, 20 = one DEI factor present, 40 = two DEI factors present, 60 = three DEI factors present, 80 = four DEI factors present, 100 = All DEI factors present). Within each subspecialty, the mean of these scores for programs was then calculated to determine a subspecialty’s completeness score.

## Results

The mean score among all programs was 12.5 out of a maximum of 100. Pediatric dermatology ranked the highest among all specialties, while Mohs ranked the lowest (Table 1). A link to the institution’s DEI homepage was the most prevalent factor, whereas information about bias training and fellowship-associated DEI webpage were the least prevalent (Table 1).

**Table 1.** Comparison of DEI content between dermatology fellowship program websites.

	Micrographic Surgery and Dermatologic Oncology (Mohs) (*N*=71)	Pediatric Dermatology (*N*=25)	Dermatopathology (*N*=56)	Total (*N*=152)
**DEI Webpage**, n (%)				
Not Present	67 (94.4%)	24 (96.0%)	56 (100.0%)	147 (96.7%)
Present	4 (5.6%)	1 (4.0%)	0 (0.0%)	5 (3.3%)
**DEI Commitment Statement**, n (%)				
Not Present	65 (91.5%)	23 (92.0%)	52 (92.9%)	140 (92.1%)
Present	6 (8.5%)	2 (8.0%)	4 (7.1%)	12 (7.9%)
**Diversity Initiatives (Summer research for med students, lectures, mentorship)**, n (%)				
Not Present	65 (91.5%)	24 (96.0%)	54 (96.4%)	143 (94.1%)
Present	6 (8.5%)	1 (4.0%)	2 (3.6%)	9 (5.9%)
**Link to the Institution’s DEI Page**, n (%)				
Not Present	54 (76.1%)	10 (40.0%)	24 (42.9%)	88 (57.9%)
Present	17 (23.9%)	15 (60.0%)	32 (57.1%)	64 (42.1%)
**Information about Bias Training**, n (%)				
Not Present	68 (95.8%)	23 (92.0%)	56 (100.0%)	147 (96.7%)
Present	3 (4.2%)	2 (8.0%)	0 (0.0%)	5 (3.3%)
**Completeness Score (out of 100)**	10.1	16.8	13.6	12.5

## Discussion

While fellowship website DEI content does not fully encompass the totality of a program’s DEI efforts, it can serve as an effective signaling method to prospective residents regarding the emphasis placed on DEI initiatives within a training program. Programs that advertise multiple DEI initiatives are likely to be viewed more favorably by under-represented minority residents and subsequently more likely to train at such institutions. Although 42.1% of fellowship program websites contained a link to the institution’s DEI webpage, most of these links were embedded in the webpages’ headers or footers, which are not unique to a fellowship’s webpage. Since these sections of a webpage are standardized, prospective residents might reasonably assume that this information was not included through the efforts of the fellowship program but rather through institution-wide initiatives. This lack of fellowship-specific DEI initiative to include information on the parts of a program’s webpage that is influenced by fellowship program leadership likely devalues a prospective resident’s perception of the importance of DEI at such program. To further underscore this point, all other DEI factors measured in this study that required direct action by fellowship leadership were included in less than 8% of all program websites. The results of this study reveal an overall lack of DEI content across all dermatology subspecialties’ webpages and represent an actionable area of improvement for fellowship directors to increase their DEI efforts, address barriers to under-represented minority recruitment, and to attract a diverse pool of applicants to their program [8]. Additionally, future studies should be conducted to assess the full impact of adding DEI content to GMEP websites. This would ideally be accomplished through survey studies of under-represented minorities assessing their perceptions of DEI content on GMEP websites as well as with studies that investigate any changes in minority residents for programs that implement high amounts of DEI content on their webpages.
